# Influence of the addition of nanohydroxyapatite to scaffolds on proliferation and differentiation of human mesenchymal stem cells: a systematic review of *in vitro* studies

**DOI:** 10.1590/1414-431X2023e13105

**Published:** 2024-01-22

**Authors:** E.L. de Melo, P.P.A.S. Cavalcanti, C.L. Pires, B.V.A. Tostes, J.M. Miranda, A.A. Barbosa, S.I.S. da Rocha, N.S. Deama, S. Alves, M.E.M.M. Gerbi

**Affiliations:** 1 Programa de Pós-graduação em Odontologia, Universidade de Pernambuco, Recife, PE, Brasil; 2 Graduação em Odontologia, Universidade de Pernambuco, Recife, PE, Brasil; 3 Programa de Pós-graduação em Química, Universidade Federal de Pernambuco, Recife, PE, Brasil; 4 Universidade Federal do Vale do São Francisco, Senhor do Bonfim, BA, Brasil

**Keywords:** Nanoparticles, Stem cell research, Cell differentiation, Nanostructures, Embryonic stem cells, Tissue scaffolds

## Abstract

One of the main challenges of tissue engineering in dentistry is to replace bone and dental tissues with strategies or techniques that simulate physiological tissue repair conditions. This systematic review of *in vitro* studies aimed to evaluate the influence of the addition of nanohydroxyapatite (NHap) to scaffolds on cell proliferation and osteogenic and odontogenic differentiation of human mesenchymal stem cells. *In vitro* studies on human stem cells that proliferated and differentiated into odontogenic and osteogenic cells in scaffolds containing NHap were included in this study. Searches in PubMed/MEDLINE, Scopus, Web of Science, OpenGrey, ProQuest, and Cochrane Library electronic databases were performed. The total of 333 articles was found across all databases. After reading and analyzing titles and abstracts, 8 articles were selected for full reading and extraction of qualitative data. Results showed that despite the large variability in scaffold composition, NHap-containing scaffolds promoted high rates of cell proliferation, increased alkaline phosphatase (ALP) activity during short culture periods, and induced differentiation, as evidenced by the high expression of genes involved in osteogenesis and odontogenesis. However, further studies with greater standardization regarding NHap concentration, type of scaffolds, and evaluation period are needed to observe possible interference of these criteria in the action of NHap on the proliferation and differentiation of human stem cells.

## Introduction

One of the biggest challenges in tissue engineering is to develop scaffolds that can simulate physiological conditions for the proliferation and differentiation of mesenchymal stem cells and contribute to tissue repair and regeneration (1-3).

The addition of nanoparticles to scaffolds has drawn attention of researchers as it favors conditions such as improved performance, increased adhesion rate, cell migration, proliferation, specific lineage differentiation, nutrient supply, and extracellular matrix deposition (3-5).

Currently, many studies have used nanoparticles of various natures in different amounts and with different methodologies, with no consensus about the need to include nanoparticles in scaffolds and the actual benefit for the proliferation and differentiation of stem cells (6-9).

Nanohydroxyapatite (NHap) is widely used because hydroxyapatite is a predominant component of calcified tissues and because it is known for its osteoconductive and osteoinductive properties (1,3-9).

Therefore, the aim of this systematic review was to assess the influence of adding NHap to scaffolds on cell proliferation and osteogenic and odontogenic differentiation of human mesenchymal stem cells.

## Methodology

### Protocol and registration

This review was performed according to the Preferred Items for Systematic Reviews and Meta-Analyses (PRISMA) statement checklist described by Moher et al. (10). The study was registered in the Open Science Framework, available at https://archive.org/details/osf-registrations-acm4u-v1.

### Research methods

Articles were individually selected by two researchers (E.L.M. and P.P.A.S.C.) in Cochrane Library, PubMed/MEDLINE, Scopus, Web of Science, ProQuest, and OpenGrey databases with no start date filtering until August/2023. Manual search was also performed in *Biomacromolecules*. Divergences were resolved by a third examiner (M.E.M.M.G.) through discussion to achieve a consensus.

The search strategy, based on the PICO criteria, was “mesenchymal stem cells AND nanohydroxyapatite AND cell proliferation AND cell differentiation OR stem cells AND nanohydroxyapatite AND scaffold AND cell proliferation AND cell differentiation”. Search strategies for each database can be found in the Supplementary Table S1.

### Eligibility criteria

Eligibility criteria were *in vitro* studies that used human mesenchymal stem cells from any type of tissue for osteogenic and odontogenic proliferation and differentiation in scaffolds containing nanohydroxyapatite. Exclusion criteria were prospective methodologies or *in vitro* studies that utilized animal stem cells, studies lacking information about the control group or intervention, studies without details regarding stem cell origin, cell culture medium, nanoparticle concentration (%), follow-up duration, evaluation methods (proliferation and differentiation), or those that did not meet the inclusion criteria described above.

### Search strategy

Studies were selected by reading the title and abstract through electronic search by two researchers (E.L.M. and P.P.A.S.C.) independently. The full reading of selected articles was carried out, and those that did not meet the inclusion criteria were excluded.

The following question was elaborated based on the PICO criteria (Population, Intervention, Comparison, and Outcome): “What is the benefit of including NHap in scaffolds in the proliferation and differentiation process of human stem cells?”. According to these criteria, the population was stem cells, the intervention was scaffolds containing nanoparticles, the comparison was scaffolds without nanoparticles, and the outcome was proliferation and differentiation.

### Bias risk

Two researchers (E.L.M. and P.P.A.S.C.) assessed the methodological quality of studies based on the evaluation framework available in the study by Marques et al. (11). Studies were evaluated for the presence of information such as cell type, culture medium, number of cell passages, culture conditions, number of cells per plate, number of experiment replications, and description of the methodology for outcome evaluation.

### Summary measures

The effect of intervention (positive or negative) was considered as a dichotomous outcome, and the amount of NHap (%) in the scaffolds, the follow-up time, and the outcome (proliferation and differentiation) were considered continuous outcomes.

### Data collection and analysis

After applying the search strategy in each database, results were transferred to the EndNote Web reference organizer and separated into folders for screening.

Qualitative data were collected and tabulated in a form previously prepared in Microsoft Word format by the team containing the necessary information for extraction by one researcher (E.L.M.) and later verified by another researcher (J.M.M.). Any divergences were resolved by a third researcher (M.E.M.M.G.) through discussion until consensus was reached.

### Additional analysis

Additional analysis was performed in the website http://www.winepi.net/ using the kappa coefficient, calculated to verify inter-examiner agreement in the selection of studies in the four databases. The kappa value was obtained by evaluating selected titles and abstracts. Inter-examiner agreement was high for Cochrane Library (90%), PubMed/MEDLINE (98.6%), Scopus (96.8%), and Web of Science (52.3%) databases.

## Results

**Figure 1:**
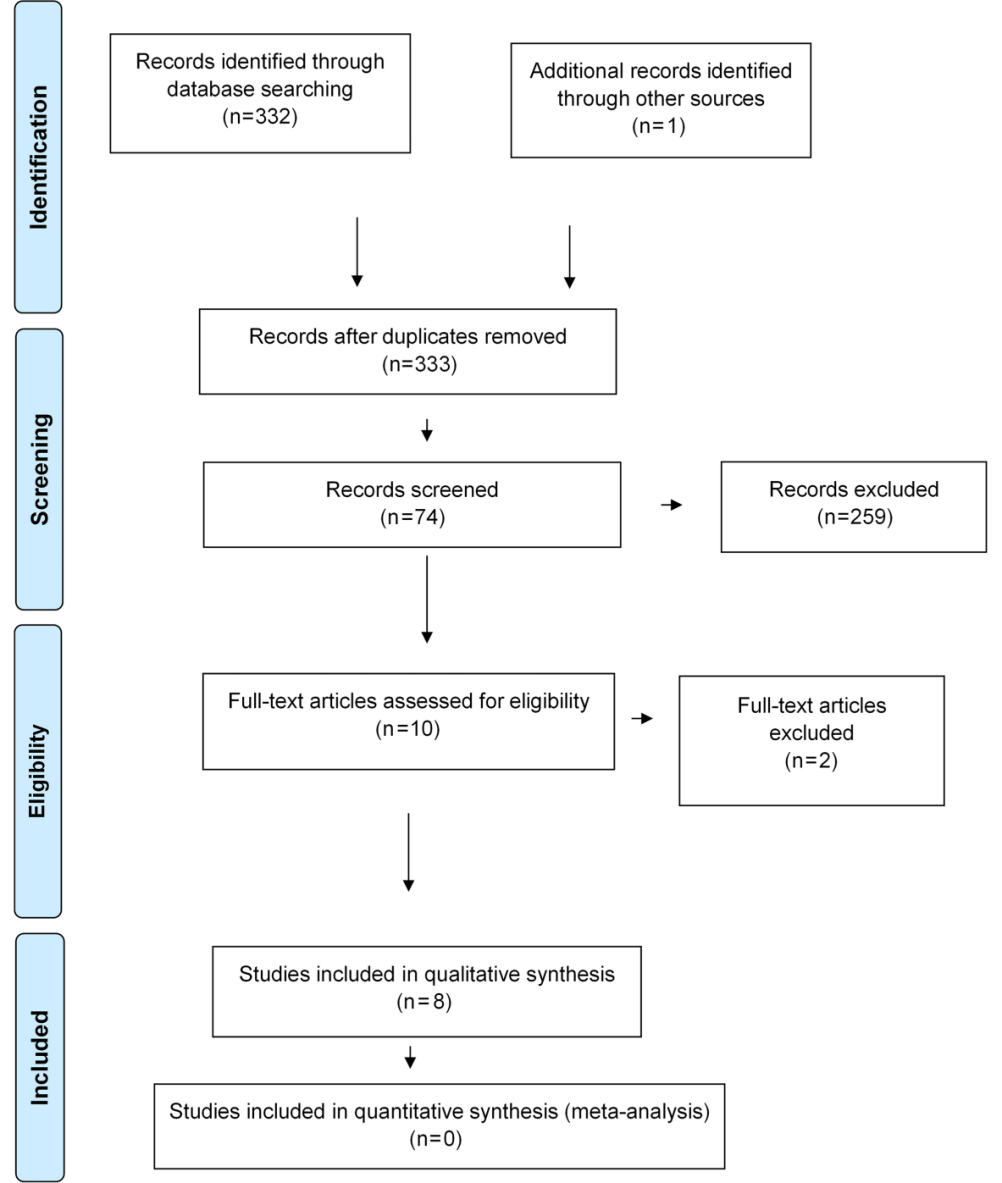
Flowchart of literature search and study selection.

A total of 333 articles were found in all databases, of which 1 was from Cochrane Library, 88 were from PubMed/MEDLINE, 105 from Scopus, 129 from Web of Science, 9 from ProQuest, 0 from OpenGrey, and 1 from the manual search in the Biomacromolecules journal. After reading titles and abstracts, 10 articles were selected for full reading (1,3,6-9,12-15). After full reading, two articles were excluded: one for working with stem cells originating from rabbits (12) and the other for not having a control group (3) (Figure 1).

In total, 8 studies were included for qualitative analysis and are summarized in Supplementary Table S2. Although the main aim of studies was to evaluate the proliferation and differentiation of stem cells in scaffolds of different compositions, in all of them, a positive effect was observed when using nanoparticles in intervention groups.

There were several sources of stem cells extraction, such as dental pulp (6), human umbilical cord (8,9), adipose tissue (13), and bone marrow (1,14,15). There was great variability in the composition of scaffolds such as poly(butylene adipate-co-terephthalate) (PBAT) (1), poly caprolactone-poly ethylene glycol-chitosan (PCEC-CS) (6), polycaprolactone gel (PCL/Gel) (7), polylactic-co-glycolic acid (PLGA) (8,13), poly(l-lactide) (PLLA) (9), chitosan/silk fibroin (CS/SF) (14), and polycaprolactone (PCL) (15),

There was significant variability in the amount (%) of NHap contained in the scaffolds, ranging from 1% (8,9) to 30% (16), in control groups, and evaluation periods. For proliferation evaluation, the shortest period found was 1 day (1,7-9,13) and the longest was 28 days (1,16). For differentiation evaluation, the shortest period found was 1 day (1,5) and the longest was 28 days (1,14).

For the proliferation/viability evaluation, 1 study used live-dead staining (13), 1 study used the hemacytometer count (3), 1 study used the MTS assay (14), 1 study used the Alamar blue assay (15), 5 studies used the MTT assay (6-9,13), 1 study used DAPI staining (6), 1 study used scanning electron microscopy (SEM) (8), and 1 study used the PicoGreen^®^ DNA quantification test (1). SEM was used in the vast majority of studies to visualize the condition of scaffolds.

For differentiation evaluation, 2 studies used qRT-PCR (6,7), 4 studies used RT-PCR (1,8,9,14), 1 study used hematoxylin and eosin staining (HE) and Masson's trichrome dye (3), 2 studies used quantification calcium (9,14), 2 studies used confocal laser scanning microscopy (14,15), 1 study used cresolphthalein complexone (9), 4 studies used Alizarin red staining (6,7,13,14), 6 studies used alkaline phosphatase (ALP) activity (1,7-9,13,14), 1 study used S stain (6), and 1 study used Von Kossa staining (7). All studies evaluated osteogenic differentiation, however only 1 study reported the gene involved in odontogenic differentiation (6).

According to bias risk analysis (Table 1), some of included studies did not report information such as amount of cell passage (1,6) and number of replicates (1,2,8,13,14). However, all studies had control and intervention groups, which are important for the evaluation of outcomes, and were therefore suitable for inclusion in this systematic review.

**Table 1:** Bias risk assessment.

Author/year	Cell type	Cell culture medium	Cell passage	Cell culture conditions	Number of plated cells per plate	Number of experimental replicates	Description of the outcome assessment methodology
Seyedjafari 2010 9	yes	yes	yes	yes	yes	NR	yes
Lai 2015 14	yes	yes	yes	yes	yes	NR	yes
Domingos 2017 15	yes	yes	yes	yes	yes	yes	yes
Hokmabad 2018 6	yes	yes	NR	yes	yes	yes	yes
Shahi 2018 8	yes	yes	yes	yes	yes	NR	yes
Arslan 2018 1	yes	yes	NR	yes	yes	NR	yes
Sattary 2019 7	yes	yes	yes	yes	yes	Yes	yes
Babilotte 2021 13	yes	yes	yes	yes	yes	NR	yes

NR: Not reported.

## Discussion

This study evaluated the influence of the addition of NHap on the proliferation and differentiation of human mesenchymal stem cells. It is important to emphasize that despite the difficulties in data standardization in systematic reviews of *in vitro* studies, such as the large variability in NHap concentration, scaffold nature, and outcome evaluation times, reviews like this one provide an overview of the contribution of nanomaterials to the field of tissue engineering.

Tissue repair strategies, such as surgery to place autologous grafts, are considered the gold standard for repairing bone defects, but have disadvantages such as postoperative pain, risk of infection, hemorrhage, and even loss of local function. Because of this, tissue engineering research has dedicated itself to developing alternative methods that are less traumatic for the patient (7,13).

Regarding the effect of NHap on cell culture, this review showed that all studies had a positive effect on intervention groups, corroborating Hokmabad et al. (6), who reported that the addition of NHap provides a suitable environment for cell proliferation and differentiation due to characteristics such as increased surface roughness, favoring the absorption of chemical species from the surrounding environment. Hydroxyapatite (HAp), Ca_10_(PO_4_)_6_(OH)_2_, is one of the members of the apatite family, Ca_10_(PO_4_)_6_(F,OH,Cl)_2_ (4,17). Since it is the main mineral component of bones and teeth, synthetic HAp stands out in the field of material science for biological applications (5,13,18,19). It is worth mentioning that HAp present in living beings generally has impurities attributed to small amounts of CO_3_^2-^ and water. According to Dorozhkin (20), biological HAp crystals are small building blocks on the scale of nanometers. Elliott et al. (21) describe that the crystals that compose bone and dentin have an approximate size of 15×40 nm, while this value in enamel is around 40×100 nm. Therefore, the use of HAp in the form of nanoparticles becomes highly relevant, since its properties are even more similar to natural particles, and it can be used for biomineralization and as a biomaterial of high biocompatibility (2,5,22-25). Furthermore, as it is an easily obtainable and inexpensive biomaterial, it attracts the interest of researchers, reduces research costs, and brings positive results.

The biological characteristics of HAp, which classify it as an excellent material for application in the medical field, have already been demonstrated in several studies, such as by Carmo et al. (26), who verified from *in vivo* tests with mice that nanostructured HAp, both carbonated and doped with Sr^2+^ ions, shows excellent results in terms of biocompatibility, bioactivity, osteoconduction, and bioreabsorption. Barbosa et al. (17) carried out hemolysis tests using erythrocytes from mice and observed that NHap presented a hemolysis degree close to 2.0%, indicating the hemocompatibility of the material. Al-Kattan et al. (27), using *in vitro* assays with human cells, obtained cell viability >80.0% for NHap concentrations up to 1000 μg/mL doped with 2.0% Eu^3+^ ions, confirming the non-toxicity of the material. In any case, further studies should be carried out with the aim of minimizing intervention in the cell while keeping the NHap concentration as low as possible.

As for the origin of stem cells, most studies used human umbilical cord. This is probably because it is easy to obtain since it is an appendage of the human body that is discarded after birth, does not have as many ethical obstacles compared to other human body sources, it is free of contamination, and contains a large amount of stem cells in the Wharton's jelly. Stem cells from human teeth, for example, can be contaminated, since extracted teeth in most cases are affected by caries microorganisms. Other sources may be difficult to acquire compared to the umbilical cord (25,26,28).

Ji et al. (3) evaluated the osteogenic differentiation of stem cells originated from human fibroblasts. Stem cells were cultivated in two types of scaffolds, one containing nanospheres and the other containing nanorods. The results showed that the presence of nanospheres significantly increased cell proliferation compared to the group with nanorods, generating a large amount of bone formation. Therefore, further studies evaluating the influence of nanoparticle morphology on stem cell proliferation and differentiation should be carried out.

The literature shows that there is no standard period for the evaluation of cell proliferation and differentiation in cultures with scaffolds. Marques et al. (11) published a systematic review on the proliferation and differentiation of stem cells, which included studies with evaluations before intervention, 5 min later, and 20, 24, 48, 72 h after intervention. In the present study, the follow-up period ranged from 1 to 28 days for both differentiation and proliferation, depending on the different methodologies adopted. Although this does not seem to affect the outcomes, it may hinder the synthesis of results for a better understanding in systematic reviews as well as replication in future *in vitro* studies. Thus, future studies should focus on establishing protocols for evaluation periods of cell culture in scaffolds.

Shahi et al. (8) found a high proliferation rate in 7 days and high ALP activity and differentiation in 21 days. The authors emphasized the expression of Osteonectin and Runx2 in cells grown in NHap-containing scaffolds. The porosity of the nanoparticle surface is considered to favor cell adhesion and proliferation, inducing bone tissue (8,24). In addition to its osteoconductivity, hydroxyapatite acts as a buffer against the acid products of polyesters in cell functions (1,2,5,23,24). This finding may explain the potentiation and acceleration of the cell proliferation process.

In osteogenic differentiation, RunX, OCN, OPN, ALP, Osteonectin, and Osteocalcin expressions were found. In odontogenic differentiation, DSSP gene expression was found, which is considered the key to odontogenic differentiation. The presence of these genes in studies involving both osteogenic and odontogenic differentiation is an expected finding.

Regarding the bias risk analysis, two studies did not report the number of passages (1,6), and four studies did not report the number of replicates of experiments. Information such as number of cell passages and replicates is extremely important for understanding and clarity in the construction and replication of studies (13,16,19,28). We recommend that this information be very clearly stated in future publications.

### Conclusion

The inclusion of NHap had a positive effect, enhancing proliferation and favoring osteogenic and odontogenic differentiation. Thus, the use of NHap in tissue regeneration is a promising alternative.

## Supplementary Material

Click here to view [pdf].
