# Supplementary material for: Influence of the addition of nanohydroxyapatite to scaffolds on proliferation and differentiation of human mesenchymal stem cells: a systematic review of in vitro studies
Source: Braz J Med Biol Res. 2024 Jan 22;57:e13105. doi: 10.1590/1414-431X2023e13105 (PMC10802233; doi:10.1590/1414-431X2023e13105)
Supplement: Supplementary file 1 [file 1414-431X-bjmbr-57-e13105-suppl.pdf]

Table S1. Results of electronic and manual search strategies per database.

Database	Search strategy	Results	Filter
PubMed	mesenchymal stem cells AND nanohydroxyapatite AND cell proliferation AND cell differentiation OR stem cells AND nanohydroxyapatite AND scaffold AND cell proliferation AND cell differentiation	88	No
Cochrane	stem-cell AND nanohydroxyapatite	1	No
Scopus	mesenchymal stem cells AND nanohydroxyapatite AND cell proliferation AND cell differentiation OR stem cells AND nanohydroxyapatite AND scaffold AND cell proliferation AND cell differentiation	105	No
Web of Science	mesenchymal stem cells AND nanohydroxyapatite AND cell proliferation AND cell differentiation OR stem cells AND nanohydroxyapatite AND scaffold AND cell proliferation AND cell differentiation	129	No
ProQuest	mesenchymal stem cells AND nanohydroxyapatite AND cell proliferation AND cell differentiation OR stem cells AND nanohydroxyapatite AND scaffold AND cell proliferation AND cell differentiation	9	Dissertations and Theses
OpenGray	stem cells AND nanohydroxyapatite	0	No

Table S2. Characteristics of included studies.

Author/ Year (Ref No.)	Stem cell origin	Cell culture medium	Amount of nanoparticle (%)	Intervention	Control	Evaluation methods	Follow-up time	Outcome		Effect of intervention
								Proliferation	Differentiation	
Seyedjafari 2010 (9)	Human umbilical cord	PLLA scaffolds	1%	PLLA + NHap scaffolds	PLLA scaffolds	SEM, MTT assay, ALP activity, RT-PCR, and calcium content assessment.	Proliferation: 1,3,5, and 7 days Differentiation: 7 and 14 days	Cell proliferation rate increased rapidly until the fifth day. After this period, there was slower proliferation in groups with scaffolds	There was high calcium content in the scaffold with nanoparticles at 14 days. The maximum ALP peak at 7 days was observed in PLLA + NHap scaffolds. The osteocalcin gene expressed high levels in PLLA + NHap scaffolds.	Positive
Lai 2015 (14)	Human bone marrow	CS/SF scaffolds	10 and 30%	CS/SF + NHap scaffolds	CS/SF scaffolds	MTS test, laser scanning confocal microscopy, ALP activity assay, alizarin red staining, calcium quantification, and RT-PCR	Proliferation and differentiation: 3, 7, 14, 21, and 28 days	The proliferation of cells showed no significant difference among groups; however, the incorporation of NHap and the concentration of embedded NHap did not affect cell viability and proliferation	At day 3 and 7–14, both CS/SF 30% NHap scaffolds exhibited significantly higher ALP concentrations compared to CS/SF/10% NHap scaffolds. Alizarin: On day 7, the alizarin red stain revealed higher intensity in CS/SF + 30% NHap scaffolds. The extent of osteogenic differentiation of cells was found to be	Positive

									positively correlated with the duration and NHap content in the scaffolds.	
Domingos 2017 (15)	Human bone marrow	PCL scaffolds	25%	PCL + NHap scaffolds	PCL scaffolds	Alamar Blue assay, ALP activity and confocal laser scanning microscope	Proliferation and differentiation: 7, 14, and 21 days	The addition of NHap enhanced the adhesion and viability of human mesenchymal stem cells	After 14 days of incubation, PCL + NHap scaffolds exhibited higher levels of ALP activity.	Positive
Hokmabad 2018 (6)	Dental pulp	PCEC-CS scaffolds	10 and 15%	PCEC-CS + NHap scaffolds	PCEC-CS scaffolds	MTT assay qRT-PCR, DAPI stain, alizarin red and S stain	Proliferation: 3, 7, and 12 days Differentiation: 21 days	At 12 days the proliferation rate in all scaffolds containing NHap was significantly higher when compared to the control group.	*BGLAP*, *BMP2*, and *DSSP* genes showed high levels of expression in scaffolds in control, and intervention groups. DSSP is a key gene for odontogenesis. Runx2 was significantly high in the PCEC-CS + NHap scaffolds, unlike the control group.	Positive
Shahi 2018 (8)	Human umbilical cord	PLGA scaffolds	1%	PLGA + NHap scaffolds	PLGA scaffolds	SEM, ALP activity and RT-PCR	Proliferation: 1, 3, 5, and 7 days Differentiation: 1, 4, 7, 14, and 21 days	Proliferation occurred in all groups, including the PLGA + NHap scaffold, until day 7.	ALP: Only in the PLGA + NHap scaffolds, a sustained ALP activity was observed up to the 13th day. Osteonectin and RUNX2 expression peaked at 7 days. Levels declined within 14 days and resumed high expression within 21 days.	Positive
Arslan 2018 (1)	Human bone marrow	PBAT scaffolds	5%	PBAT + NHap scaffolds	PBAT scaffolds	Quant-iT™ PicoGreen™ DNA Test, SEM, ALP, RT-PCR	Proliferation and differentiation: 1, 7, 14, and 28 days	At 7, 14, and 28 days of culture, the intervention group showed significantly higher amount of DNA.	At 7 days, the ALP activity in the intervention group was significantly higher than in the control group. In 28 days, it remained constant. At 28 days, the amount of calcium was high in all groups.	Positive
Sattary 2019 (7)	Adipose tissue	PCL/Gel scaffolds	20%	PCL/Gel + NHap scaffolds	PCL/Gel scaffolds	SEM, EDX, TEM, FTIR, MTT assays, Alkaline Phosphatase, Von Kossa stain, alizarin red stain, and qRT-PCR	Proliferation: 1, 4, and 7 days Differentiation: 7, 14, and 21 days	In 4 days, proliferation was significantly increased in all groups	ALP activity was statistically significant at 14 days between groups. In PCR, the COLL I, BGLAP, ALP, and RUNX2 levels increased in all groups.	Positive

Babilotte 2021 (13)	Adipose tissue	PLGA scaffolds	5 and 10%	PLGA scaffolds with NHap	PLGA scaffold	MTT assay, Live-Dead staining, ALP, and alizarin red	Proliferation: 1, 2, 3, 7,14, and 21 days Differentiation: 3,7,14, and 21 days	Cells proliferated on both scaffolds. By day 14, the cells began to form a bridge-like structure and colonized the pores	The first detected ALP activity was around day 14. The staining appeared more intense with PLGA-NHap scaffolds compared to PLGA scaffolds.	Positive

PBAT: poly(butylene adipate-co-terephthalate); PCEC-CS: poly caprolactone-poly ethylene glycol-chitosan; PCL: polycaprolactone; PLGA: polylactic-co-glycolic acid; PLLA: poly(L-lactide); CS/SF: chitosan/silk fibroin; SEM: scanning electron microscopy; ALP: alkaline phosphatase; RT-PCR: reverse transcription-polymerase chain reaction test; NHap: nanohydroxyapatite; PLGA-Hap: polylactic-co-glycolic acid/hydroxyapatite.
